# Extracellular Vesicles in Mycobacteria and Tuberculosis

**DOI:** 10.3389/fcimb.2022.912831

**Published:** 2022-05-27

**Authors:** Carolina Mehaffy, Joan M. Ryan, Nicole A. Kruh-Garcia, Karen M. Dobos

**Affiliations:** Colorado State University, Department of Microbiology, Immunology and Pathology, Fort Collins, CO, United States

**Keywords:** exosomes, extracellular vesicle, mycobacteria, tuberculosis, proteomics

## Abstract

Tuberculosis (TB) remains a public health issue causing millions of infections every year. Of these, about 15% ultimately result in death. Efforts to control TB include development of new and more effective vaccines, novel and more effective drug treatments, and new diagnostics that test for both latent TB Infection and TB disease. All of these areas of research benefit from a good understanding of the physiology of *Mycobacterium tuberculosis* (Mtb), the primary causative agent of TB. Mtb secreted protein antigens have been the focus of vaccine and diagnosis research for the past century. Recently, the discovery of extracellular vesicles (EVs) as an important source of secreted antigens in Mtb has gained attention. Similarly, the discovery that host EVs can carry Mtb products during *in vitro* and *in vivo* infection has spiked interest because of its potential use in blood-based diagnostics. Despite advances in understanding the content of Mtb and Mtb-infected host extracellular vesicles, our understanding on the biogenesis and role of Mtb and host extracellular vesicles during Mtb infection is still nascent. Here, we explore the current literature on extracellular vesicles regarding Mtb, discuss the host and Mtb extracellular vesicles as distinct entities, and discuss current gaps in the field.

## Introduction

It has been almost 140 years since the *Mycobacterium tuberculosis* bacillus was first described, yet TB remains a public health crisis. Before the current pandemic, TB was considered the number one cause of death due to an infectious agent. Today, TB sits as the second leading infectious cause of death world-wide behind COVID-19 (World Health Organization, 2021). While there are diagnostic and surveillance methods and programs, treatments, and even a vaccine, this bacterium infects over 10 million people and claims the lives of 1.5 million individuals each year (World Health Organization, 2021). Although Mtb can infect anyone, it disproportionally impacts vulnerable groups like those in poverty, migrants, refugees, prisoners, and people with HIV. Drug resistance complicates the situation, with multidrug-resistant and extensively drug-resistant cases representing up to 4% of new cases annually (Kurz et al., 2016; Martini et al., 2018). Unfortunately, the World Health Organization’s End TB Strategy has experienced a setback in the world of COVID-19, with TB deaths rising for the first time in 2020 in over a decade. Access to care has been severely impacted by the pandemic, and public health spending efforts for TB control have reverted to levels previously seen in 2016 (World Health Organization, 2021).

Extracellular vesicles (EVs) are secreted by nearly every cell and organism across all three domains of life (Gill et al., 2019). In 1946, Chargaff and West reported the first observation of EVs—then referred to as pro-coagulant platelet-derived particles—which became known as “platelet dust” by Wolf in 1967 (Chargaff and West; Wolf, 1967). Anderson described matrix vesicles during bone calcification in 1969, and in the following decade several other mammalian cell types were noted to produce these structures (Anderson, 1969). In 1983 the first ultrastructural study demonstrating the release of vesicles by multi-vesicular bodies confirmed that production of some types of vesicles is a regulated phenomenon (Harding et al., 1983; Pan and Johnstone, 1983). Fast forward to today, and we now know that mammals, plants, parasites, arthropods, fungi, archaea, and bacteria produce EVs.

EVs are membrane bound structures ranging from 20-1000 nm in diameter (reviewed in Gould and Raposo, 2013). While specific contents are influenced by the cell of origin and its physiologic state, EVs carry protein, lipids, carbohydrates, nucleic acids, and metabolites (Gill et al., 2019). These vesicles have the capacity to transfer biomolecules between cells, influencing physiological function in recipient cells (Cheng and Schorey, 2019). The contents, size, and membrane composition are heterogeneous and change depending on the state of the cellular source and environmental conditions. Different classification systems have arisen in order to classify EVs based on things like cellular origin, size and morphology, biogenesis, and function (Gould and Raposo, 2013).

In the context of Mtb infection and disease, host and Mtb EVs have emerged as a focus of study. Host EVs can carry Mtb molecules such as DNA, mRNA, lipids and proteins and are being exploited as potential biomarkers for TB and LTBI (Kruh-Garcia et al., 2014; Lv et al., 2017; Mehaffy et al., 2017; Dahiya et al., 2019; Mehaffy et al., 2020). Host EVs released by infected cells during Mtb infections are also being studied because of their immunomodulatory properties (Bhatnagar and Schorey, 2007; Giri et al., 2008).

Mtb EVs are proposed to play different roles in the physiology of the bacilli. Since host and Mtb EVs are difficult to differentiate with current processing methods, some authors hypothesize that Mtb molecules identified in EVs during *in vivo* infection may be the result of a mixed population of host and Mtb EVs. Based on our previous studies as well as those published elsewhere, we discuss Mtb EVs and host EVs as different entities with distinct protein cargo. In this review we will explore the current literature, current knowledge gaps, and future perspectives of Mtb EVs and host EVs in the context of Mtb infection with an emphasis on their protein cargo.

## Mycobacterial Extracellular Vesicles

Outer membrane vesicles (OMV), a subset of EVs specific to Gram-negative bacteria, have been a popular field of study since 1966 (Knox et al., 1966). In contrast, the production of EVs by Gram-positive and mycobacteria has just recently gained attention. Because Gram-positive and mycobacteria differ in their cell envelope composition, it was surprising to discover that vesicles can be released despite their complex cell wall structure and today the mechanisms of their EV biogenesis have yet to be completely elucidated (Brown et al., 2015). The first report of extracellular vesicle production by mycobacteria was published in 2007, in which *M. ulcerans* biofilm formation and its role in transmission were described. Incidentally, scanning electron microscopy (SEM) imaging revealed vesicle association with the biofilm. This observation led to the first purification of mycobacterial EVs and immunoprecipitation of these EVs from infected mouse tail tissues (Marsollier et al., 2007). Not long after this initial publication, production of EVs by many other environmental and pathogenic mycobacterial species, including *Mycobacterium tuberculosis*, was demonstrated (Prados-Rosales et al., 2011). Additional studies have confirmed EV production in other mycobacterial species, including the pathogenic *Mycobacterium avium* subspecies *paratuberculosis* (MAP) and *Mycobacterium avium* subspecies *hominissuis* (MAH) (Chiplunkar et al., 2019; Palacios et al., 2019)

### Composition of Mycobacterial EVs

In their 2011 exploration of mycobacterial EVs, Prados-Rosales et al. began to describe the composition and role of Mtb EVs (Prados-Rosales et al., 2011). To show that production was occurring from live cells, C-acetate radiolabeled *Mycobacterium bovis* BCG were sub-cultured and given five days to replicate before vesicle isolation. The C-acetate label was detected only in vesicles from live cells and not in vesicle-like aggregates formed from heat killed cells. In addition to visualizing vesicles from Mtb H37Rv and BCG, they looked at a variety of mycobacteria and found that slow and fast growers, virulent, avirulent, pathogenic, and environmental species produce EVs that are roughly the same size (20-300 nm) and exhibit a predominantly closed membrane morphology with both unilaminar and bilayered structures. Using 2D thin layer chromatography (TLC) and matrix-assisted laser desorption/ionization (MALDI) time of flight (TOF) mass spectrometry (MS), the group investigated the lipid composition of Mtb and BCG EVs. Polar lipids including phosphatidylinositol, phosphatidylinositol mannosides (PIMs), phosphatidylethanolamine (PE), and cardiolipin, accompanied by the lipoglycan Lipoarabinomannan (LAM), but not α-glucan, were found to make up the EV membrane. This points to the inner cytoplasmic membrane as the origin of mycobacterial EVs (Prados-Rosales et al., 2011). Contrastingly, a later study found that EVs from *M. avium* (MAH) contain lipids from the outer layers of the cell wall (Chiplunkar et al., 2019). A comprehensive comparison of mycobacterial EV lipid composition across species, strains, and culture conditions would help clarify this contradiction.

Proteomic analysis of Mtb EVs consistently demonstrates lipoprotein enrichment, with LpqH and LprG strongly represented. Lipoproteins made up to 8% of the identified proteins in one study, while they comprise only 1-2% of proteins in the whole Mtb genome (Lee et al., 2015). Functional categorization of EV proteins interrogated by mass spectrometry identify cell wall, membrane function, and intermediate metabolism and respiration as the predominant functional groups. Additionally, many identified proteins contribute to host-pathogen interactions, with several known toll-like receptor 2 (TLR-2) ligands consistently enriched (Prados-Rosales et al., 2011; Lee et al., 2015). Transmission electron microscopy (TEM) of immunogold antibody labeled EVs confirmed the presence of LpqH and LprG in the membrane of Mtb EVs. LAM was also seen in these preparations in association with the EVs (Prados-Rosales et al., 2011).

To date there has only been one mention of nucleic acid in Mtb EVs, and it was unpublished data indicating that DNA was detected in purified, intact Mtb EVs (Prados-Rosales et al., 2011). In MAH EVs, double stranded DNA has been reported in the lumen and on the surface with the majority present on the surface (Chiplunkar et al., 2019). There was not enough DNA material to warrant sequencing based on the goals of this study. When comparing RNA content between EVs produced by infected macrophage cells and those from Mtb cell culture, very little RNA was detected in the bacterial EVs (Singh et al., 2015). Specific profiles of carbohydrates, metabolites, and nucleic acids from mycobacterial EVs have yet to be defined.

### Mycobacterial EV Functions

Defining the functions of mycobacterial EVs remains an active area of investigation. The first reported role of virulence mediation was identified in *M. ulcerans* EVs. Lipid analysis revealed the presence of mycolactone in the vesicles. When applied to mouse bone marrow-derived macrophages (BMM) and COS cells (non-phagocytic fibroblast-like cell line from monkey kidney tissues), the vesicles were more cytotoxic than an equal amount of purified mycolactone (Marsollier et al., 2007). *M. smegmatis* EVs carry TlyA, a protein previously shown to have hemolytic activity, which remains functional and active in the vesicles and is also produced by Mtb (Kumar et al., 2015). EVs allow for the transport of molecules in a protected manner, resulting in concentrated amounts of virulence factors or other important molecules, overcoming problems related to solubility or dilution through diffusion.

Mtb EVs also play a role in nutrient acquisition—specifically the uptake of iron, which is a scarce resource in the host. Mtb cultured in limited iron environments produced EVs with mycobactin, an efficient sideophore that is normally envelope-bound. The EVs produced in low iron can rescue an Mtb mutant incapable of making siderophores like mycobactin. The mycobactin rich EVs can also rescue wild type Mtb from severe iron deprivation (Prados-Rosales et al., 2014b). The protein composition of MAH vesicles produced in minimal medium versus a metal mix medium (meant to mimic the phagosome) varies, with 52 proteins in common, 211 specific to the metal mix and 150 specific to the minimal medium (Chiplunkar et al., 2019). This further confirms that mycobacterial EVs are involved in response to nutrient availability in the environment.

Immunomodulation is a more complex function attributed to mycobacterial EVs. As previously mentioned, mycobacterial EVs from pathogenic species carry a variety of TLR-2 agonists including LpqH, LprG, LprA, PhoS1, and LAM among others (Prados-Rosales et al., 2011). TLRs recognize pathogen-associated molecular patterns (PAMPs) and initiate signal transduction pathways that regulate the expression of cytokines, chemokines, and type I IFNs for innate and adaptive immune activation. TLR-2 signal transduction cascades in macrophages result in the induction of a CD4+ T cell response (Oliveira-Nascimento et al., 2012). Prolonged TLR-2 activation, however, is immunosuppressive, leading to the production of immunosuppressive cytokines like IL-10 and ultimately inhibition of MHC-II antigen presentation (Harding and Boom, 2010). The initial induction of TLR-2 starts CD4+ T cell activation, which promotes granuloma formation, but is not enough to eliminate the bacteria as the effects of prolonged TLR-2 signaling come in to play. Mtb TLR-2 agonists, including LpqH, LprG, LprA, LAM, and others, were shown to cause prolonged TLR-2 signaling (Noss et al., 2001; Tapping and Tobias, 2003; Gehring et al., 2004; Pecora et al., 2006). This effect is also seen when cells are exposed to Mtb EVs both *in vitro* and *in vivo*, demonstrating that vesicles serve as a concentrated delivery system for these agonists providing some insight into how the prolonged signaling occurs (Prados-Rosales et al., 2011).

Mtb EVs also influence immune cells through mechanisms other than TLR-2 signaling in macrophages. Direct exposure to Mtb EVs inhibits T cell activation, demonstrated by reduced IL-2 production and reduced T cell proliferation (Athman et al., 2017). Mtb EVs induce partial anergy during primary stimulation of naïve T cells and they inhibit effector T cells in a transient manner. Temporary inhibition of effector T cells may facilitate local inhibition of Th1 responses at the site of infection without causing a systemic inhibition of T cell response (Athman et al., 2017). In contrast to limiting antigen presentation in macrophages, exposure of mouse dendritic cells (DC) to Mtb EVs induced MHC-I, MHC-II, and CD86 expression, resulting in DC maturation and antigen presentation to Ag85B-specific CD4+ T cells (Jurkoshek et al., 2016). Although it is clear that Mtb EVs have multiple functions, clarifying conflicting roles in the host-pathogen interaction remains an important avenue of inquiry.

### Mycobacterial EV Biogenesis

Arguably the biggest mysteries remaining in mycobacterial EV biology are genetic regulation and specific mechanisms for biogenesis and release. As previously mentioned, early studies of Mtb and BCG EV lipid composition point to the cell membrane as the origin point for mycobacterial EVs (Prados-Rosales et al., 2011). A subsequent study by the same group confirmed that no lipids from the outer membrane were detected in Mtb EVs, regardless of the concentration of iron in the media (Prados-Rosales et al., 2014b). The authors indicated that additional studies to understand vesicle synthesis may help explain the lack of outer membrane lipids in Mtb EVs. Contrastingly, outer membrane lipids were detected in EVs from MAH (Chiplunkar et al., 2019). It is possible that individual species have different mechanisms of EV biogenesis. Production of different EV subpopulations by specific mycobacteria species or differences in culturing process or EV enrichment method may also influence the composition or ability to detect lipids from different subcellular compartments.

Mycobacterial EV production kinetics and composition vary based on the status of the cell of origin, indicating that biogenesis of EVs is a regulated process. Mycobacterial EV release kinetics during culture is dependent on the mycobacterial species. *M. bovis* and Mtb have similar production patterns (Palacios et al., 2019), while MAP is notably different with lower EV production, a lack of unilamellar vesicles, and slightly larger diameter. Under iron limitation, Mtb produces significantly more EVs (of the same size and morphology, but differing siderophore content) than in an iron-rich environment (Prados-Rosales et al., 2014b). Similarly, MAH EVs vary in protein composition based on the metal nutrients present in culture (Chiplunkar et al., 2019). Exposure to sub-inhibitory concentrations of INH triggers an increase in Mtb EV production (Gupta et al., 2020). Approximately 10% of the time when *M. smegmatis* divides, it happens asymmetrically resulting in very short (< 2 µm) and very long (> 7 µm) cells (Vijay et al., 2017). This occurs regardless of the growth medium, indicating this is part of the normal population dynamics. Interestingly, these short cells contain more lipids and have a higher density of EVs on the surface compared to the normal and very long cells when imaged by SEM and TEM. The short cells are more sensitive to INH, rifampicin, H_2_O_2_, and acidification (Vijay et al., 2017). It seems possible the increase in EV production on these cells may result in higher cell envelope permeabilities and result in increased sensitivity to the environment.

The first experiments to reveal potential mechanisms of mycobacterial EV biogenesis involved a transposon mutant of Mtb H37Rv with a null allele insertion for rv0431 that was successfully complemented back, resulting in overexpression (Rath et al., 2013). This mutant was previously shown to grow normally in culture but exhibited attenuation in mice (Beaulieu et al., 2010). Deficiency in this protein (now named VirR for “vesiculogenesis and immune response regulator”) results in similar protein concentration released in the culture filtrate proteins (CFP) fraction and normal cell wall integrity, but a significant increase in lipid release in the CFP and EV production per cell than the wild type (WT) (Rath et al., 2013). The VirR mutant was not more susceptible to acidification, oxidative stress or nitrosative stress, and did not have changes in the other mycobacterial protein export pathways (SecA1, SecA2, TAT or T7S). Interestingly, there was more LpqH and SodC in the EVs of the mutant strain, which reverted upon complementation. Treatment of BMM with these EVs resulted in more TNF- α and IL-12 p40 production compared to treatment with WT EVs. Overall, this study indicates that VirR regulates EV generation and dampens the activation of macrophages, interfering with the host’s ability to control Mtb proliferation, which is at least in part mediated by TLR-2. It is also likely that VirR is part of a higher-order complex with at least three likely binding partners (Rv1488, Rv0383c, and LpqH) (Rath et al., 2013).

Another gene seemingly involved in Mtb EV biogenesis is rv3371, which encodes for a triacylglycerol synthase (TGS) (Rastogi et al., 2017). This gene plays a role in the deliberate metabolic slowdown of Mtb after establishing infection in the host (Baek et al., 2011). Expression of rv3371 in *M. smegmatis* increases the triacylglycerol levels, changes the surface from dry and rough to smooth and wet, increases the presence of bud-like structures on the surface, and results in shorter cells (Rastogi et al., 2017). Together, these changes suggest that TGS functions in the cell wall and expression may be linked to replication events. Expression of rv3371 in Mtb H37Rv increases during late log phase and through stationary phase. It also increases under hypoxia, nutrient starvation, and nitrosative and oxidative stress. All of these conditions are phases of slow or stopped growth. The authors found that iron deprivation caused a significant increase in rv3371 transcription, but no increase was seen for any other TGS or the dormancy survival regulon (DosR). Although deletion of rv3371 in H37Rv does not change growth rate or colony morphology, there is apparent cell wall alteration based on changes observed after staining cells that were grown in conditions known to increase rv3371 expression. Interestingly, loss of rv3371 leads to an apparent decrease in EV secretion as observed by scanning electron microscopy, which can be complemented back to normal levels. This apparent decrease in EV secretion is also seen in low-iron conditions, and complementation results in a hypervesiculation phenotype in low-iron media (Rastogi et al., 2017). This gene is essential for Mtb survival in the mouse. It is also required for growth arrest *in vitro*, but not in normal culture conditions. Based on this, and the fact that its transcripts have been found in the human lung granuloma, the authors suggest that investigating the role of rv3371 in *in vivo* persistence could determine if this gene is a good drug target. While promising, it is important to clarify that the observations related to rv3371 and vesicle formation need to be validated by extracting EVs from both WT and rv3371 KO and performing a systematic analysis, including vesicle quantification and characterization by Nanoparticle Tracking Analysis.

A different mechanism that may be involved in Mtb EV production relates to the phosphate-specific transport (Pst) system. Pst interacts with the two-component SenX3-RegX3 system based on inorganic phosphate availability. This interaction results in an increase of ESX-5 protein secretion (Elliott and Tischler, 2016). Deletion of pstA1 (a transmembrane component of the Pst system) results in activation of SenX3-RegX3, independent of inorganic phosphate availability. This mutant has a RegX3-dependent increase in esx5 transcription and hypersecretion of two ESX-5 substrates associated with Mtb EVs: PPE41 and EsxN (Tischler et al., 2013; Elliott and Tischler, 2016). Further proteomic characterization showed that the ΔpstA1 mutant secretes significantly more LpqH and PstS1, which is caused by a significant increase in EV production; up to fifteen times more vesicles than WT (White et al., 2018). This increase in vesiculation was ultimately shown to be independent of ESX-5, but requires Reg3X. Interestingly, the ΔpstA1 mutant’s hypervesiculation is not dependent on VirR, but ΔpstA1ΔvirR produced 4 fold more EVs than ΔpstA1 and 63 fold more EVs than ΔvirR, which suggests that the mechanisms driving EV production in these strains synergize (White et al., 2018).

Most recently, investigation into the role of dynamin-like proteins (DLPs) for Mtb EV biogenesis was released as a pre-print (not peer reviewed) in 2020 (Gupta et al., 2020). DLPs are guanosine triphosphate hydrolase enzymes that mediate membrane fusion and fission in both prokaryotes and eukaryotes. A soluble DLP, IniA, was shown to deform membranes, contribute to membrane fission, membrane remodeling, maintenance of plasma membrane integrity, and notably, contribute to isoniazid-resistance based on studies with *M. smegmatis* (Wang et al., 2019). As a member of the LytR-CpsA-Psr protein family, VirR is also likely involved in cell envelope integrity since other members of the family include enzymes that transfer glycopolymers from membrane-linked precursors to cell envelope proteins or peptidoglycan (Kawai et al., 2011). Examination of both ΔvirR and iron-limited Mtb revealed changes in the cell envelope, with loss of VirR causing increased thickness and iron limitation causing thinning (Gupta et al., 2020). In both strains, the iniBAC operon and [Fe-S] assembly genes were induced as compared to WT Mtb. Since the metabolic stress from VirR inactivation and iron limitation is not identical, the upregulated genes in both conditions likely converge on a shared function. Indeed, deletion of iniA results in decreased vesiculation, but protein secretion concentrations are maintained as compared to the WT (Gupta et al., 2020). The impacts on vesiculation from ΔiniA could not be rescued by iniA or iniC individually but could be rescued by a plasmid with the entire WT iniBAC operon. Additionally, EVs from ΔiniA cannot rescue cells from iron-limitation even though the strain makes enough mycobactin/carboxymycobactin in normal media for itself (Gupta et al., 2020). The ΔiniA strain replicated similarly to WT during macrophage infection, but had seemingly decreased EV production based on reduction of EVs containing bacterial components in the cell culture. These experiments further confirm a link between cell envelope alterations and Mtb EV release, demonstrate convergence of several factors influencing Mtb EV release, and provide a potential mechanism for targeting vesicle production *in vivo* (Gupta et al., 2020).

Understanding mycobacterial EV composition, functions, and secretion mechanisms is important for several potential applications including drug development, biomarker-based diagnostics, and vaccine production. To date, there are not completely null Mtb mutants for vesiculation, which strongly indicates this function as essential for bacterial viability (Coelho and Casadevall, 2019). Some of the most successful medications for tuberculosis target cell-envelope related processes, including INH and ethambutol (among others) (Jackson et al., 2013). Since EV release undoubtedly involves cell-envelope alterations—and given the ties between vesiculation and environmental stress like what the bacteria encounters in the host—defining various vesicle biogenesis mechanisms may provide new targets for antitubercular drugs.

Because the composition of mycobacterial EVs varies based on the bacterial species and includes highly immunogenic biomolecules in pathogenic species, use as a diagnostic serology test for evidence of infection and even discrimination between infection by closely related species is an active area of investigation. In 2013, Ziegenbalg et al. discovered that serum from TB patients reacts strongly and significantly to Mtb EVs (Ziegenbalg et al., 2013). The IgG responses were significantly higher in sputum smear positive versus smear negative cases and absent in healthy, BCG vaccinated, tuberculin skin test positive serum samples. Paratuberculosis, caused by MAP, impacts up to 50% of European and North American bovine herds (McAloon et al., 2019). There is a vaccine, but it does not provide complete protection and can cause interference with tests for bovine tuberculosis caused by *M. bovis* so there is a need to easily distinguish among bovine TB, paratuberculosis infection, and paratuberculosis vaccination. Mycobacterial EVs may provide the answer since sera from *M. bovis* infected cows recognize LpqH and other proteins from Mtb EVs, while sera from healthy, MAP infected, and MAP vaccinated cows did not (Palacios et al., 2019). Interestingly, the EVs from *M. bovis* and MAP did not demonstrate good diagnostic serology capacity for any of these groups.

Mtb EVs were demonstrated to be strong stimulators of the inflammatory immune response in mice, with increased and rapid granuloma formation in the lungs of mice administered H37Rv EVs through the trachea (Prados-Rosales et al., 2011). Subsequently, subcutaneous administration of mycobacterial EVs alone and in combination with BCG-vaccination indicated that these vesicles may serve as effective vaccine components (Prados-Rosales et al., 2014a). Both BCG and Mtb EV immunization elicit a strong humoral and cellular response to membrane and cell wall components of Mtb. Vaccination using Mtb EVs without adjuvant protected mice against H37Rv infection through a very strong Th1 mediated response. The Mtb EV vaccinated mice were protected as well as the regular BCG immunization group; however, BCG immunization did not generate as strong of a Th1 activation. Protection was not seen in mice vaccinated with BCG derived EVs. Additionally, Mtb EV administration following BCG immunization boosted the immunity afforded by BCG-vaccination alone, resulting in a more diverse antibody response. Unfortunately, there were challenges with reproducibility within the study whereby only two of the three studies of Mtb EVs alone as a vaccine component demonstrated protection (Prados-Rosales et al., 2014a). Based on this study, use of Mtb EVs as part of a vaccine requires a better understanding of the source of variability. Additionally, if the consistent preparation of Mtb EVs is not achievable, then understanding what parts of the EVs mediate the protective immune response may allow for the generation of artificial EVs carrying those components. In order to move from preclinical to clinical proof of concept studies, active component characterization is of paramount importance as well as developing a straightforward manufacturing and quality control process (Sarmiento et al., 2019).

## Host Extracellular Vesicles in the Context of Mtb Infection

As previously mentioned, essentially every cell generates extracellular vesicles. This makes many (if not all) human and animal biofluids a complex mixture of vesicles from hundreds of cell types. Biofluids are home to natural flora and pathogens whose vesicles may also contribute to the global population for that fluid. The exact contribution of microorganisms to circulating extracellular vesicles is not currently known, including potential differences between extracellular and intracellular pathogens and commensal organisms. In the previous sections, we reviewed the current knowledge of mycobacterial EVs during *in vitro* culture. Host-derived vesicles in the context of *Mtb* infection have also been a focus of study. It is possible that host derived vesicles may contain a mix of mycobacterial EVs, especially during *in vitro* culture of cell monolayers. However, during *in vivo* infection, host EVs isolated from sterile biofluids such as blood and serum are likely to represent only host derived EVs. Here we will explore the current status of the literature on host EVs during Mtb infection.

EVs as biomarkers of TB disease has emerged as a potential tool to help move away from sputum-based diagnostics to more accessible biofluids like blood. This is vital because sputum-based diagnostics are difficult to use for children and individuals with HIV/AIDS. Further, sputum-based diagnostics are only useful to diagnose TB disease. Diagnosis of Latent TB infection relies on diagnostics based on host immune responses and thus, also has limitations in individuals with HIV/AIDS and other conditions resulting in immunosuppression. In 2014, our group developed a discovery approach utilizing *in vitro* macrophage infections, the mouse and guinea pig animal models, and corroboration in human samples to profile the protein cargo of host EVs during Mtb infection. Ultimately, we were able to identify peptides corresponding to 33 unique Mtb proteins in EVs isolated from serum of TB patients (Kruh-Garcia et al., 2014). Interestingly, the majority of the proteins identified were secreted antigens (**Table 1**) which have not been shown to be enriched in Mtb EVs, supporting the notion that EVs isolated from in the context of Mtb infection are of host and not bacterial origin. A side-to-side proteomic characterization of host EVs isolated from patients with LTBI and TB, and compared to mycobacterial EVs isolated by the same protocols and produced in different conditions including those likely to be encounter in the host such as iron and oxygen limiting conditions remains an important aspect for future work by our group. In further studies, we optimized the mass spectrometry methods (Multiple Reaction Monitoring-Mass Spectrometry, MRM-MS) and demonstrated more than 90% accuracy in distinguishing TB positive versus TB negative (Mehaffy et al., 2017). Peptides from Cfp2, Mpt32, Mpt64, and BfrB were the most consistently identified. We have also shown that host EVs may carry Mtb proteins or peptides during LTBI. Our MRM-MS detected Mtb peptides in host serum EVs of at least 95% of individuals with LTBI (Mehaffy et al., 2020). A peptide from GlnA1 was detected in the majority of the serum EV samples. As mentioned previously, based solely on protein composition, these studies almost certainly represent assays for human origin EVs. In addition, it is highly unlikely that Mtb EVs travel beyond the lung environment and circulate in the blood. This is especially true when the infection is dormant, as with LTBI. In addition to monitoring Mtb proteins in EVs as an avenue for biomarkers of infection, the host EV proteome changes significantly following infection (Diaz et al., 2016; Diaz, 2017). Simultaneously evaluating host and Mtb proteins in serum EVs may allow for a multi-faceted approach to defining TB infection status.

**Table 1:** Comparison of Mtb protein content of host EVs and Mtb EVs.

	Discovery in purified host EVs^1^			Targeted proteomics human EVs^1,2^	MtbEvs^3^
	MO	Mouse	Human	TB	
Rv0009|ppiA				x	
Rv0066c|icd2		x		x	
Rv0125|pepA		x		x	
Rv0129c|Ag85c	x	x		x	x
Rv0350|DnaK		x	x	x	
Rv0363c|Fba		x		x	x
Rv0440|groEL2		x			
Rv0934|PstS1	x	x	x		x
Rv1270c|lprA	x	x		x	
Rv1469|CtpD		x	x	x	
Rv1827|GarA|Cfp17	x	x		x	
Rv1837c|GlcB	x	x	x		
Rv1860|Apa|Mpt32	x	x		x	x
Rv1886c|Ag85b	x	x		x	x
Rv1908c|KatG	x	x		x	
Rv1926c|Mpt63	x	x		x	x
Rv1932|Cfp20|Tpx	x	x		x	
Rv1980c|Mpt64	x	x		x	
Rv2031c|HspX	x	x	x	x	x
Rv2220|GlnA1	x	x		x	
Rv2244|AcpM	x	x		x	x
Rv2376c|Cfp2	x	x		x	
Rv2626c			x		
Rv2780|Ald	x	x		x	
Rv2878c|Mpt53	x	x		x	x
Rv3248c|SahH	x	x		x	
Rv3418c|GroES	x	x		x	
Rv3441c|MrsA			x	x	
Rv3803c|Mpt51|fbpD	x	x		x	x
Rv3804c|Ag85a	x	x		x	x
Rv3841|BfrB	x	x		x	
Rv3874|CFP10				x	
Rv3875|EsxA|Esat6	x				

Only proteins validated in host EVs are included. Mtb EVs are enriched with TLR2 agonist lipoproteins which are not reflected in this table.

References: 1. Kruh-Garcia et al, 2014; 2. Mehaffy et al., 2017, 3. Lee et al., 2015.

While Mtb EV biogenesis is the biggest unknown from the bacterial physiology perspective, the fate of Mtb EVs *in vivo* remains one of the most challenging questions from the host-pathogen interaction lens. Several studies have visually demonstrated through electron microscopy that mycobacteria release vesicles while intracellular (Prados-Rosales et al., 2011; Majlessi et al., 2015; Chiplunkar et al., 2019). Athman et al. (2015) provided strong evidence for the presence of Mtb EVs in Mtb-infected macrophage culture supernatants. Using triple immunogold labeling, they visualized MHC-II, CD-9, and Mtb components (anti-Mtb gold antibody) on vesicles. Vesicles in which Mtb components were evident, appear to lack MHC-II and CD-9 (very occasionally, a mix of host and Mtb markers were seen on the same vesicles) (Athman et al., 2015). After attempting to separate the vesicle populations and evaluating their impacts on human embryonic kidney cells and mouse macrophages, they conclude that “the TLR-2 agonist and proinflammatory activities attributed to EVs released from Mtb-infected cells is derived from Mtb EVs and not from host cell-derived EVs” (Athman et al., 2015).

In contrast with Athman et al. (2015), there is evidence that host EVs, rather than Mtb EVs, are the dominant EV type containing Mtb proteins enriched from infected cell cultures. Smith et al. (2015) found that inhibiting ubiquitination in Mtb-infected macrophages significantly reduced Mtb protein levels in subsequently released EVs (Smith et al., 2015). This is important because ubiquitin-interacting motifs are found on subunits of the endosomal sorting complex required for transport (ESCRT) machinery (Katzmann et al., 2001). ESCRTs are involved with human exosome biogenesis (Vietri et al., 2020). In fact, when inhibiting ESCRT, the production of EVs by uninfected macrophages was reduced by over 80% (Smith et al., 2015). Mtb proteins were strongly detected after enrichment of ubiquinated proteins from Mtb-infected macrophage EVs by immunoprecipitation. The authors found that ubiquination of GroES and HspX was required for those proteins to be detected in Mtb-infected macrophage EVs (Smith et al., 2015). While mycobacteria have a process called pupylation that is somewhat similar to ubiquination for protein degradation, the eukaryotic ubiquitin tag is completely different from the pupylation tag (Akhter and Thakur, 2017). Therefore, the most plausible conclusion for GroES and HspX presence in the Mtb-infected macrophage EVs is through the host ESCRT pathway (Smith et al., 2015).

Another study with Mtb-infected mice demonstrated similar conclusions using Rab27a, a protein involved in exosome biogenesis. Macrophages from mice deficient in Rab27a released 80% fewer EVs than macrophages from WT mice following Mtb infection (Smith et al., 2017). The Rab27a deficient Mtb-infected macrophages released less EVs that also had fewer Mtb proteins. These EVs failed to elicit pro-inflammatory responses in macrophages. Combined with the decrease in Mtb proteins in EVs following ubiquitination inhibition (Smith et al., 2015), this supports the hypothesis that the dominant Mtb protein-containing EVs from infected cell cultures are actually host derived. Interestingly, the levels of LpqH present in EVs from the Rab27a deficient and WT macrophages after Mtb infection remained the same, which might support the presence of a minor population of Mtb EVs (Smith et al., 2017).

Authors from the ubiquination and Rab27a studies suggest that the conclusions reached by Athman et al. (2015) were premature (Schorey et al., 2021). Schorey et al. (2021) state that “this conclusion is based on the detection of LAM-positive vesicles lacking the exosomal markers CD9 and CD63; however recent data for exosomes and other [human] EVs indicate a significantly greater heterogeneity in vesicle composition than previously appreciated, with classic exosome markers such as CD9 and CD63 being present on only a subset of exosomes (Kowal et al., 2016)” (Schorey et al., 2021). In addition to exosome diversity regarding classical markers such as CD9 and CD63, it is important to note that The immunofluorescence microscopy labeling in the Athman et al. study was performed with polyclonal anti-Mtb, which “detects LAM and LM, although its specificity is not limited to these lipoglycans” (Athman et al., 2015). It is therefore impossible to determine if the antibody is staining non-LAM/LM Mtb biomolecules or host glycolipids (through recognition of conserved glycerophosphotidylinositol anchors), although LAM and LM are expected to be the dominantly stained antigens. Without a clear answer to the fate of Mtb EVs in mixed culture or the origin of Mtb proteins in EVs from infected cell cultures, the following discussion assumes that EVs from cell cultures infected with mycobacteria or cultures exposed to mycobacterial EVs *via* CFP contain a mix of host and bacterial vesicles. Therefore, functions observed in these studies cannot be specifically ascribed to EVs of either origin based on the information provided.

### Role of EVs Released From Infected Cells During Mtb Infection

Extracellular vesicles from mycobacteria-infected cells contribute to the innate immune response by promoting the recruitment and activation of immune cells. In a TLR-2 dependent manner, EVs from *M. avium*-infected macrophages stimulate a proinflammatory response in mouse BMMs (Bhatnagar and Schorey, 2007). Similarly, EVs from Mtb- or BCG-infected macrophages induce bone marrow-derived DC maturation and activation (Giri et al., 2008). MAP-infected macrophage EVs carry mycobacterial proteins ESAT-6, Mpt63, SodA, Mpt51, and Ag85. Treating naïve macrophages with these EVs increases their rate of phagocytosis and secretion of proinflammatory cytokines but does not induce apoptosis and necrosis like exposure to the actual MAP bacteria (Wang et al., 2015). This latter fact supports potential use of these MAP-infected macrophage EVs in vaccines or therapeutics. Along these lines, intranasal injection of mice with EVs from BCG- or Mtb-infected macrophage cultures induced proinflammatory cytokine production and resulted in the recruitment of neutrophils and macrophages to the lungs (Bhatnagar et al., 2007).

EVs from Mtb-infected macrophages have also been shown to activate endothelial cells (Li et al., 2018). This results in greater expression of chemokine receptors, cell adhesion molecules, and release of chemokines. The same endothelial cell responses are observed with exposure to EVs from the serum of Mtb-infected mice. Additionally, BMMs can migrate through endothelial cells that have been exposed to EVs from infected cells, but not when exposed EVs from uninfected cells (Li et al., 2018).

Mycobacterial RNA is present in EVs from Mtb-infected macrophages, which depends on functional SecA2 in the bacteria (Cheng and Schorey, 2019). These EVs induce a type I IFN response in macrophages based on the RIG-I/MAVS-dependent foreign RNA detection pathway. Activation of this pathway is required for the EVs released by Mtb-infected macrophages to induce restriction of Mtb replication in neighboring macrophages (Cheng and Schorey, 2019). Finally, treatment of Mtb-infected macrophages with EVs from Mtb-infected neutrophils increases superoxide anion production and autophagy in the macrophage, leading to increased bacterial killing (Alvarez-Jiménez et al., 2018). Together, these studies demonstrate that EVs from a variety of *Mycobacterium*-infected innate immune cells incite further innate immune activation both *in vitro* and *in vivo*.

Adaptive immune activation can also be facilitated by EVs from *Mycobacterium*-infected cells. EVs from BCG- and Mtb-infected macrophages can present peptide-MHC-II complexes to T cells (Ramachandra et al., 2010). However, this process is more effective in the presence of other antigen presenting cells because they are more efficient at stimulating memory T cells than naïve T cells (Ramachandra et al., 2010). Mice treated with EVs from BCG-infected macrophages produce CD4+ and CD8+ T cells in the spleen, lung, and mediastinal lymph nodes (Giri et al., 2008). These T cells produce IFN- γ upon *ex vivo* stimulation with BCG [119]. These findings were reproduced using EVs from macrophages treated with Mtb CFP rather than mycobacterial infection (Giri et al., 2010). Interestingly the *ex vivo* stimulation of splenic cells from Mtb CFP vaccinated mice with EVs from CFP-treated macrophages resulted in a more robust cytokine response, suggesting that enrichment of the molecules in the vesicles provides a more efficient trafficking and antigen presentation to the splenic cells (Giri et al., 2010). Also, EVs from CFP treated macrophages were shown to protect mice when administered intranasally, at a level equivalent to BCG vaccination, following low-dose aerosol Mtb infection (Cheng and Schorey, 2013).

**Figure 1:**
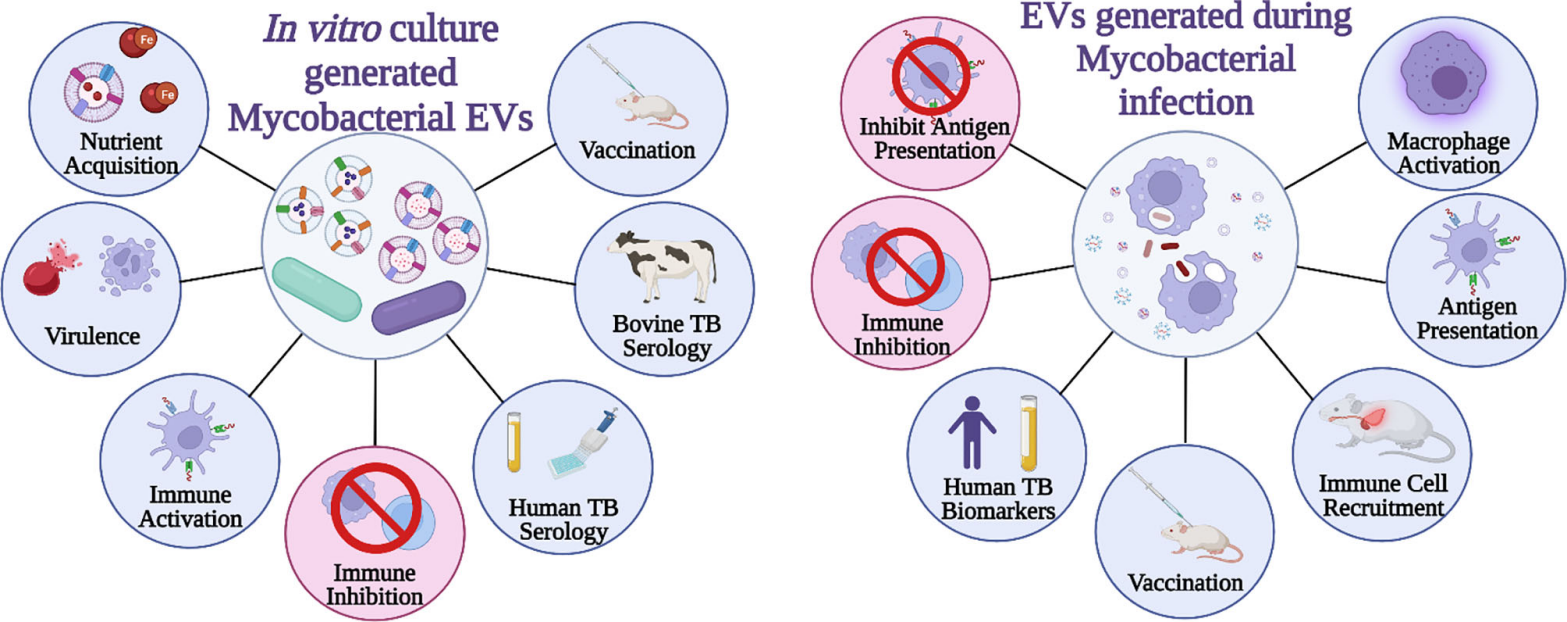
Summary of mycobacterial EVs. General functions and applications of EVs **(A)** generated strictly by mycobacteria and **(B)** those generated by mycobacterium-infected cells. Created with BioRender.com.

In contrast to immune activation, EVs from mycobacterial infected cells also demonstrate immune suppression. Macrophage MHC-II and CD-64 expression in response to IFN-γ is partially inhibited following treatment with EVs from Mtb-infected macrophages, which is dependent on TLR2 (Singh et al., 2011). Additionally, EVs from Mtb-infected macrophages inhibit CD4+ T cell activation similarly to, but more strongly than, LAM alone. This resulted in reduced IL-2 production and T cell proliferation (Athman et al., 2017). Without the ability to efficiently and completely separate mycobacterial EVs from eukaryotic EVs and/or the ability to block EV production by Mtb or eukaryotic cells, determining which EVs are causing which responses throughout the course of infection is incredibly challenging. Attempts to separate the two vesicle populations suggest a density gradient may work to achieve this (Athman et al., 2015), however, there were aspects of the experimental design that were problematic. First, the vesicles were loaded at the top of the gradient while flotation gradients by upward displacement are more appropriate for separation (Lötvall et al., 2014; Théry et al., 2018). Additionally, the reported density for the bacterial EVs, which likely have a higher lipid to protein ratio, is higher than the human vesicles in the study and still in the reported range for EVs of human origin (Athman et al., 2015). It is impossible to confirm complete separation of human and Mtb EVs based on the data provided. Further proteomic analysis of the density gradient-enriched populations, as suggested by Schorey et al. (2021), would help answer this question (Schorey et al., 2021). The functions specifically attributed to mycobacterial EVs from mycobacterial culture verses EVs generated during *in vitro* or *in vivo* infection is provided in **Figure 1**.

## Discussion of Current Gaps and Future Directions

### Mycobacterial EV’s Biogenesis

To date, the structural origin of mycobacterial EVs appears contradictory, with some studies suggesting the cytoplasmic membrane as their point of origin and other studies indicating a role for the bacilli’s cell wall. In addition, mycobacterial EVs are found as unilamellar or bilayered which suggests different structural origins and perhaps different biogenesis pathways. Improvements to EVs purification, including separation of EVs subpopulation by size and a comprehensive comparison of mycobacterial EV lipid and protein composition across species, strains, and culture conditions would help clarify the contradictory and ambiguous findings reported to date.

### Mycobacterial EV’s Content

Proteomic analysis have shone a light to the protein cargo of mycobacterial EVs. Identification of protein in Mtb EVs seems to vary among different studies (Prados-Rosales et al., 2011; Lee et al., 2015). Given that Mtb EV composition varies based on the environment (Prados-Rosales et al., 2014b), it is important to understand what contents are present under standard culture conditions versus various states of stress. However, despite discrepancies in the number of proteins identified, there appears to be a consensus for the enrichment of TLR2 agonists such as lipoproteins in mycobacterial EVs. Similarly, lipid profiling has allowed the identification of major lipid classes in mycobacterial EVs. However, limited knowledge is available for the composition of carbohydrates, metabolites, and nucleic acids of mycobacterial EVs. Identification of small molecules could help advance the understanding of the *Mycobacterium* metabolic status under different conditions and could help identify additional roles for Mtb EVs in host-pathogen interactions with non-classical T cells.

### Mycobacterial EV’s Function

Mycobacterial EVs seem to play a role in nutrient transport and iron scavenging. Both of these roles are likely to be important during *in vivo* infection where both of these resources are scarce. In addition, mycobacterial EVs are shown to have immunomodulatory abilities in innate and adaptive immune response. Mycobacterial EVs are enriched with TLR2 agonists which help explain some of the host-pathogen interaction findings, however additional studies focused on understanding how mycobacterial EVs are generated and circulated during intracellular infection will help identify any other potential roles of these vesicles in host-pathogen interactions.

### Host EV’s and Mtb Infection

EVs carrying Mtb molecules have been demonstrated during *in vitro* and *in vivo* infection. Additional profiling of EVs generated from different phagocytic cells such as alveolar macrophages, dendritic cells and neutrophils could help improve the understanding of the role of EVs in innate immunity. In addition, identification of host markers in EVs from these different cell types could also help in the development of separation techniques (i.e. immunoaffinity) that allow purification of EVs in complex biofluids and enrichment of host EVs likely carrying Mtb molecules. Host EVs carrying Mtb molecules have been mostly demonstrated in serum. One study demonstrated the presence of LAM and Cfp10 in EVs isolated from urine (Dahiya et al., 2019). Analysis of mycobacterial molecules in host EVs isolated from other biofluids such as saliva, sputum and BAL may be enriched in host EVs originating from the infection sites before they enter circulation and their analysis may provide a better understanding of the role of EVs during infection.

In 2014 the Minimal Information for Studies of Extracellular Vesicles (MISEV) was published to help combat variations in rigor and reproducibility for EV studies (Lötvall et al., 2014). While this publication and the subsequent update in 2018 focus heavily on eukaryotic EVs, many of the challenges noted and the principals discussed apply to EVs of all origins (Théry et al., 2018). Variation in EV composition based on the recovery or enrichment protocol has been well documented for eukaryotic EVs and extends to mycobacterial EVs as well [133–137] (Kalra et al., 2013; Cvjetkovic et al., 2014; van Deun et al., 2014; Zonneveld et al., 2014; Dauros Singorenko et al., 2017). In fact, technical reproducibility issues within density gradient separation are physically visible with *M. smegmatis* EVs, and the difference in protein, particle, and RNA recovery between enrichment techniques is quite significant (Dauros Singorenko et al., 2017). Technical inconsistencies combined with variations in the culture conditions for Mtb EV generation may be influencing the data collected and conclusions drawn regarding Mtb EVs. Improvements to, and standardization of purification methods may help in elucidating some of the current gaps highlighted above and may also help further differentiation between host and Mtb EVs during *in vivo* infections.

## Author Contributions

CM and JR contributed equally to the drafting and writing of this review. CM worked on the initial outline for the concept of this review. NK-G supported the research to JR, provided critical review and edit. KD supported the research to CM, JR, and NK-G, developed the concept with CM, and further with JR and NK-G, and provided initial review, edit and final review and edit. All authors contributed to the article and approved the submitted version.

## Funding

Funding for this article was provided by Colorado State University to KD.

## Conflict of Interest

The authors declare that the research was conducted in the absence of any commercial or financial relationships that could be construed as a potential conflict of interest.

## Publisher’s Note

All claims expressed in this article are solely those of the authors and do not necessarily represent those of their affiliated organizations, or those of the publisher, the editors and the reviewers. Any product that may be evaluated in this article, or claim that may be made by its manufacturer, is not guaranteed or endorsed by the publisher.
